# Multicontaminant air pollution in Chinese cities

**DOI:** 10.2471/BLT.17.195560

**Published:** 2018-02-05

**Authors:** Lijian Han, Weiqi Zhou, Steward TA Pickett, Weifeng Li, Yuguo Qian

**Affiliations:** a State Key Laboratory of Urban and Regional Ecology, Research Centre for Eco-Environmental Sciences, Chinese Academy of Sciences, Beijing 100085, China.; b Cary Institute of Ecosystem Studies, Millbrook, United States of America.; Correspondence to Weiqi Zhou (email: wzhou@rcees.ac.cn).

## Abstract

**Objective:**

To investigate multicontaminant air pollution in Chinese cities, to quantify the urban population affected and to explore the relationship between air pollution and urban population size.

**Methods:**

We obtained data for 155 cities with 276 million inhabitants for 2014 from China's air quality monitoring network on concentrations of fine particulate matter measuring under 2.5 μm (PM_2.5_), coarse particulate matter measuring 2.5 to 10 μm (PM_10_), nitrogen dioxide (NO_2_), sulfur dioxide (SO_2_) and ozone (O_3_). Concentrations were considered as high, if they exceeded World Health Organization (WHO) guideline limits.

**Findings:**

Overall, 51% (142 million) of the study population was exposed to mean annual multicontaminant concentrations above WHO limits – east China and the megacities were worst affected. High daily levels of four-contaminant mixtures of PM_2.5_, PM_10_, SO_2_ and O_3_ and PM_2.5_, PM_10_, SO_2_ and NO_2_ occurred on up to 110 days in 2014 in many cities, mainly in Shandong and Hebei Provinces. High daily levels of PM_2.5_, PM_10_ and SO_2_ occurred on over  146 days in 110 cities, mainly in east and central China. High daily levels of mixtures of PM_2.5_ and PM_10_, PM_2.5_ and SO_2_, and PM_10_ and SO_2_ occurred on over  146 days in 145 cities, mainly in east China. Surprisingly, multicontaminant air pollution was less frequent in cities with populations over 10 million than in smaller cities.

**Conclusion:**

Multicontaminant air pollution was common in Chinese cities. A shift from single-contaminant to multicontaminant evaluations of the health effects of air pollution is needed. China should implement protective measures during future urbanization.

## Résumé

**Objectif:**

Étudier la pollution de l'air par plusieurs contaminants dans les villes chinoises, quantifier la population urbaine touchée, et analyser la relation entre la pollution de l'air et la taille de la population urbaine.

**Méthodes:**

Nous avons recueilli des données auprès du réseau de surveillance de la qualité de l'air de la Chine sur les concentrations de matières particulaires fines mesurant moins de 2,5 μm (PM_2.5_), de matières particulaires grossières mesurant de 2,5 à 10 μm (PM_10_), de dioxyde d'azote (NO_2_), de dioxyde de soufre (SO_2_) et d'ozone (O_3_) dans 155 villes comptant un total de 276 millions d'habitants pour l'année 2014. Les concentrations étaient considérées comme élevées lorsqu'elles dépassaient les limites indicatives fixées par l'Organisation mondiale de la Santé.

**Résultats:**

En tout, 51% (142 millions) de la population étudiée a été exposée à des concentrations annuelles moyennes de plusieurs contaminants supérieures aux limites définies par l'OMS, l'est de la Chine et les mégalopoles étant les plus touchés. En 2014, des concentrations quotidiennes élevées de mélanges de quatre contaminants – PM_2.5_, PM_10_, SO_2_ et O_3_, et PM_2.5_, PM_10_, SO_2_ et NO_2_ – ont été mesurées pendant 110 jours dans de nombreuses villes principalement situées dans les provinces du Shandong et du Hebei. Des concentrations quotidiennes élevées de PM_2.5_, PM_10_ et SO_2_ ont été mesurées pendant 146 jours dans 110 villes principalement situées à l'est et au centre de la Chine. Des concentrations quotidiennes élevées de mélanges de PM_2.5_ et PM_10_, PM_2.5_ et SO_2_, et PM_10_ et SO_2_ ont été mesurées pendant 146 jours dans 145 villes principalement situées à l'est de la Chine. Étonnamment, la pollution de l'air par plusieurs contaminants était moins fréquente dans les villes comptant plus de 10 millions d'habitants que dans les villes de plus petite taille.

**Conclusion:**

La pollution de l'air par plusieurs contaminants s'est révélée courante dans les villes chinoises. Les évaluations des effets de la pollution de l'air sur la santé ne doivent plus tenir compte d'un seul contaminant, mais de plusieurs contaminants. Il est essentiel que la Chine mette en application des mesures de protection dans le cadre de l'urbanisation à venir.

## Resumen

**Objetivo:**

Investigar la contaminación atmosférica con múltiples contaminantes en las ciudades chinas, cuantificar la población urbana afectada y explorar la relación entre la contaminación del aire y el tamaño de la población urbana.

**Métodos:**

Se obtuvieron datos de 155 ciudades con 276 millones de habitantes de 2014 de la red de seguimiento de la calidad del aire de China sobre las concentraciones de partículas finas que miden menos de 2,5 μm (PM_2,5_), partículas gruesas que miden 2,5 a 10 μm (PM_10_), dióxido de nitrógeno (NO_2_), dióxido de azufre (SO_2_) y ozono (O_3_). Las concentraciones se consideraron altas si superaban los límites de las directrices de la Organización Mundial de la Salud (OMS).

**Resultados:**

En general, el 51% (142 millones) de la población del estudio estuvo expuesta a unas concentraciones de múltiples contaminantes medias anuales por encima de los límites de la OMS: el este de China y las megaciudades se vieron más afectadas. Hasta en 110 días en 2014, hubo altos niveles diarios de mezclas de cuatro contaminantes de PM_2,5_, PM_10_, SO_2_ y O_3_, y PM_2,5_, PM_10_, SO_2_ y NO_2_ en muchas ciudades, principalmente en las provincias de Shandong y Hebei. Hubo altos niveles diarios de PM_2,5_, PM_10_ y SO_2_ durante más de 146 días en 110 ciudades, principalmente en el este y el centro de China. Hubo altos niveles diarios de mezclas de PM_2,5_ y PM_10_, PM_2,5_ y SO_2_, y PM_10_ y SO_2_ durante más de 146 días en 145 ciudades, principalmente en el este de China. Sorprendentemente, la contaminación del aire con múltiples contaminantes fué menos frecuente en las ciudades con poblaciones de más de 10 millones que en las ciudades más pequeñas.

**Conclusión:**

La contaminación del aire con múltiples contaminantes es habitual en las ciudades chinas. Se necesita un cambio en las evaluaciones de los efectos de la contaminación del aire en la salud de un solo contaminante a múltiples contaminantes. China debería implementar medidas de protección en futuras urbanizaciones.

## ملخص

**الغرض:**

الاستقصاء بشأن تلوث الهواء بفعل الملوثات المتعددة في الصين، وقياس مدى تأثر سكان الحضر، واستكشاف العلاقة بين تلوث الهواء وحجم سكان الحضر.

**الطريقة:**

حصلنا على بيانات لعام 2014 من شبكة رصد نوعية الهواء الصينية وذلك لعدد 155 مدينة يسكنها 276 مليون نسمة، وتدور البيانات حول تركيزات الجسيمات الناعمة الدقيقة التي تقل أقطارها عن 2.5 ميكرومتر (PM _2.5 _)، والجسيمات الخشنة التي يتراوح قطرها من 2.5 إلى 10 ميكرومتر (PM _10 _)، وثنائي أكسيد النيتروجين (NO _2 _)، وثنائي أكسيد الكبريت (SO _2 _)، والأوزون (O _3 _). واعتبرت التركيزات مرتفعة في حالة تجاوزها لحدود المبادئ التوجيهية لمنظمة الصحة العالمية.

**النتائج:**

كان ‏51% (142 مليون نسمة) من السكان الذين شملتهم الدراسة عرضة بوجه عام لتركيزات من الملوثات المتعددة يتجاوز متوسط حجمها السنوي الحدود المقررة لمنظمة الصحة العالمية، وكان شرق الصين والمدن الكبرى هي الأماكن الأكثر تضررًا.

وقد وُجدت مستويات مرتفعة يومية من مخلوطين من أربعة ملوثات يتمثلان في PM _2.5 _ مع PM _10 _ وSO _2 _ وO _3 _، فضلاً عن PM _2.5 _ مع PM _10 _ وSO _2 _ وNO _2 _، وذلك لمدة أقصاها 110 أيام في عام 2014 في العديد من المدن ولاسيما في شاندونغ ومقاطعة خبي.

وقد وجدت مستويات مرتفعة يومية من PM _2.5 _ وPM _10 _ وSO _2 _ لما يزيد عن 146 يومًا وذلك في 110 مدن، ولاسيما في شرق الصين ووسطها. ووجدت مستويات مرتفعة يومية من مخاليط PM _2.5 _ مع PM _10 _، وPM _2.5 _ مع SO _2 _، وPM _10 _ مع SO _2 _ لما يزيد عن 146 يومًا وذلك في 145 مدينة على الأخص في شرق الصين ووسطها. ومن المثير للدهشة أن تلوث الهواء الناتج عن الملوثات المتعددة كان أقل شيوعًا في المدن التي يزيد عدد سكانها عن 10 ملايين نسمة عنها في المدن الأصغر.

**الاستنتاج:**

يشيع تلوث الهواء الناتج عن الملوثات المتعددة في المدن الصينية. وثمة حاجة إلى تغيير التقييمات من تقييمات الملوثات المنفردة إلى تقييمات للملوثات المتعددة للوقوف على الآثار الصحية الناتجة عن تلوث الهواء. وينبغي على الصين أن تقوم بتنفيذ التدابير الوقائية خلال التطور الحضري المستقبلي.

## 摘要

**目标:**

调查中国城市多污染物空气污染情况，以量化受影响的城市人口，并探讨城市人口规模与空气污染之间的关系。

**方法:**

我们从中国空气质量监测网获得了 2014 年 155 个城市 2.76 亿居民的数据，包括 2.5 μm (PM_2.5_) 以下的细颗粒物质、2.5 至 10 μm (PM_10_) 的粗颗粒物质、二氧化氮 (NO_2_)、二氧化硫 (SO_2_) 和臭氧 (O_3_) 浓度。如果其浓度超过世界卫生组织指南上限，会认为其过高。

**结果:**

总体而言，51% （1.42 亿）被研究人群暴露在高于世界卫生组织规定的年平均浓度的多污染物空气污染中——华东地区和特大城市受到的影响最大。在许多城市，主要集中在山东省和河北省，2014 年中有 110 天，PM_2.5_、PM_10_、SO_2_、O_3_ 和 PM_2.5_、PM_10_、SO_2_ 和 NO_2_ 四种污染混合物的日均含量过高。在 110 个城市中，主要集中在华东和华中地区，PM_2.5_、PM_10_、SO_2_ 日均含量过高的天数超过 146 天。在 145 个城市中，主要集中在华东地区，PM_2.5_ 和 PM_10_、PM_2.5_ 和 SO_2_、PM_10_ 和 SO_2_ 日均含量过高的天数超过 146 天。令人惊讶的是，多污染物空气污染在 1000 万以上人口城市的发生频次比较小规模城市要少得多。

**结论:**

多污染物空气污染在中国城市很常见。评估空气污染对健康的影响需要从单一污染物向多污染物转变。中国应该在未来城市化进程中采取保护措施。****

## Резюме

**Цель:**

Изучить многокомпонентное загрязнение воздуха в городах Китая, дать количественную оценку доли городского населения, находящегося под воздействием загрязненного воздуха, и изучить взаимосвязь между загрязнением воздуха и численностью городского населения.

**Методы:**

Мы получили данные 155 городов с 276 миллионами жителей за 2014 год из сети мониторинга качества воздуха в Китае по концентрациям тонкодисперсных частиц размером менее 2,5 μм (ТЧ_2,5_), крупнодисперсных частиц размером от 2,5 до 10 μм (ТЧ_10_), двуокиси азота (NO_2_), диоксида серы (SO_2_) и озона (O_3_). Концентрации считались высокими, если они превышали предельные величины, рекомендуемые Всемирной организацией здравоохранения (ВОЗ).

**Результаты:**

В целом 51% исследуемой популяции (142 миллиона человек) подвергался воздействию среднегодовых концентраций многокомпонентных загрязнений, которые превышали рекомендуемые ВОЗ пределы, — в наибольшей степени такому воздействию подверглись Восточный Китай и мегаполисы. В 2014 году во многих городах, главным образом в провинциях Шаньдун и Хэбэй, до 110 дней наблюдались высокие среднесуточные уровни четырехкомпонентных смесей ТЧ_2,5_, ТЧ_10_, SO_2_ и O_3_ и ТЧ_2,5_, ТЧ_10_, SO_2_ и NO_2_. Высокий среднесуточный уровень ТЧ_2,5_, ТЧ_10_ и SO_2_ наблюдался в течение 146 дней в 110 городах, главным образом в Восточном и Центральном Китае. Высокие среднесуточные уровни смесей ТЧ_2,5_ и ТЧ_10_, ТЧ_2,5_ и SО_2_, ТЧ_10_ и SО_2_ наблюдались в течение 146 дней в 145 городах, главным образом в Восточном Китае. Неожиданным было то, что многокомпонентное загрязнение воздуха встречалось реже в городах с населением более 10 миллионов человек, чем в небольших городах.

**Вывод:**

Многокомпонентное загрязнение воздуха было распространено в городах Китая. При оценке воздействия загрязнения воздуха на здоровье необходим переход от однокомпонентного к многокомпонентному загрязнению. Китай должен принять защитные меры во время будущей урбанизации.

## Introduction

Air pollution in cities is a major concern worldwide, irrespective of a country’s level of development. In high-income countries, air quality has improved substantially since the 1970s; however, the adverse health effects of exposure to relatively low-level pollution remains a public concern.^1^ In contrast, air quality in some middle- and low-income countries, such as China and India, has seriously deteriorated.^2^ Before the 1920s, the main cause of urban air pollution in high-income countries was the rapid spread of coal-fired industry during the second phase of the Industrial Revolution. The major contaminants produced by coal combustion are particulate matter and sulfur dioxide (SO_2_). After the1920s, a new source of air pollution emerged with the widespread use of the automobile, which emits particulate matter, nitrogen dioxide (NO_2_), lead and other contaminants. However in some middle- and low-income countries, e.g. China, the development of coal-fired industries and increased automobile use have overlapped, which has resulted in the emission of a complex mix of air contaminants.^3^^,^^4^

Most studies of the health effects of air pollution have focused on individual contaminants, such as particulate matter, NO_2_, SO_2_, ozone (O_3_) and carbon monoxide, with each considered to have an independent impact.^5^^–^^7^ However, in reality the urban atmosphere is never confronted with a single contaminant but is actually exposed to a complex mix of different contaminants at varying times of the day and year. Consequently, people are more likely to be exposed to a mixture of contaminants than to a single substance, the resultant impact on human health can be highly varied.^8^ For instance, some contaminants (e.g. NO_2_ and O_3_) affect the respiratory system, some (e.g. particulate matter) affect the circulatory system and cause heart disease and others (e.g. SO_2_) affect the skin and mucous membranes. Although few epidemiological studies have looked at the combined effect of several air contaminants, it can be assumed that they will have an impact on different parts of human body. For example, the combination of NO_2_ and particulate matter pollution will affect both respiratory and cardiovascular systems.^5^^,^^6^ As it can lead to these complex conditions, exposure to multicontaminant air pollution is important and should be quantified, especially in rapidly urbanizing developing countries where mixtures of contaminants are common.^4^^,^^9^

Previous research has paid particular attention to understanding how specific contaminants affect public health in developing countries. Although important, this approach may underestimate the actual impact of urban air pollution on public health. In fact, there have been calls for a shift from a single-contaminant to a multicontaminant approach to countering the health effects of air pollution.^5^ The aims of this study were: (i) to document the mixture of air contaminants in Chinese cities both annually and diurnally; (ii) to determine the proportion of the urban population affected by multicontaminant air pollution; and (iii) to investigate the relationship between the size of the urban population and the frequency of occurrence of high levels of multicontaminant air pollution.

## Methods

We obtained data on air quality for 155 cities (including all 31 provincial capitals and 124 major prefectural cities) from China's urban air quality monitoring network, which reports concentrations of air contaminants under the newly upgraded ambient air quality standard GB3095–2012. For this study, we used hourly concentrations of fine particulate matter less than or equal to 2.5 μm in diameter (PM_2.5_), coarse particulate matter with a diameter between 2.5 and 10 μm (PM_10_), NO_2_, SO_2_ and O_3_ for the whole of 2014. To assess pollution levels and their potential impact on public health, we used guideline values for annual and daily ambient air quality provided by the World Health Organization (WHO; Table 1).^10^ We averaged hourly concentrations to obtain annual means for all contaminants, 24-hour means for PM_2.5_, PM_10_ and SO_2_ and 8-hour means for O_3_. For the NO_2_ concentration, we retained the hourly values. Finally, we determined how frequently annual and daily multicontaminant air pollution due to various combinations of three, four and five contaminants (Table 2; available at: http://www.who.int/bulletin/volumes/96/4/17-195560) exceeded the values in Table 1 for individual substances. We obtained the size of the population in each of the 155 cities, as reported in the 2010 census, from the National Bureau of Statistics of China.^11^ In total, these cities accounted for 41.2% of China's urban population in 2010.

**Table 1:** WHO guideline values on ambient air quality, 2016^10^

Contaminant	Annual limit	Daily limit
PM_2.5_	10 μg/m^3^ annual mean	25 μg/m^3^ 24-hour mean
PM_10_	20 μg/m^3^ annual mean	50 μg/m^3^ 24-hour mean
NO_2_	40 μg/m^3^ annual mean	200 μg/m^3^ 1-hour mean
SO_2_	ND	20 μg/m^3^ 24-hour mean
O_3_	ND	100 μg/m^3^ 8-hour mean

ND: not determined; NO_2_: nitrogen dioxide; O_3_: ozone; PM_2.5_: fine particulate matter less than or equal to 2.5 μm in diameter; PM_10_: coarse particulate matter with a diameter between 2.5 and 10 μm; SO_2_: sulfur dioxide; WHO: World Health Organization.

**Table 2:** Combinations of contaminants evaluated, air pollution study, China, 2014

No. of contaminants	Combinations of air contaminants	
	Annual concentrations evaluated	Daily concentrations evaluated
**Five**	N/A	PM_2.5_, PM_10_, NO_2_, SO_2_ and O_3_
**Four**	N/A	(i) PM_2.5_, PM_10_, NO_2_ and O_3_; (ii) PM_2.5_, PM_10_, SO_2_ and O_3_; (iii) PM_2.5_, PM_10_, NO_2_ and SO_2_; (iv) PM_2.5_, O_3_, NO_2_ and SO_2_; and (v) PM_10_, O_3_, NO_2_ and SO_2_
**Three**	PM_2.5_, PM_10_ and NO_2_	(i) PM_2.5_, PM_10_ and O_3_; (ii) PM_2.5_, O_3_ and NO_2_; (iii) PM_2.5_, O_3_ and SO_2_; (iv) PM_2.5_, PM_10_ and NO_2_; (v) PM_2.5_, PM_10_ and SO_2_; (vi) PM_10_, SO_2_ and NO_2_; (vii) PM_2.5_, O_3_ and NO_2_; (viii) PM_10_, O_3_ and NO_2_; (ix) PM_10_, O_3_ and SO_2_; and (x) NO_2_, O_3_ and SO_2_
**Two**	(i) PM_2.5_ and PM_10_; (ii) PM_2.5_ and NO_2_; and (iii) PM_10_ and NO_2_	(i) PM_2.5_ and PM_10_; (ii) PM_2.5_ and O_3_; (iii) PM_2.5_ and NO_2_; (iv) PM_2.5_ and SO_2_; (v) PM_10_ and O_3_; (vi) PM_10_ and NO_2_; (vii) PM_10_ and SO_2_; (viii) O_3_ and NO_2_; (ix) O_3_ and SO_2_; and (x) NO_2_ and SO_2_

N/A: not applicable; NO_2_: nitrogen dioxide; O_3_: ozone; PM_2.5_: fine particulate matter less than or equal to 2.5 μm in diameter; PM_10_: coarse particulate matter with a diameter between 2.5 and 10 μm; SO_2_: sulfur dioxide.

The main variable of interest in our study was exposure to a high level of multicontaminant air pollution, which was defined as occurring when the concentration of a contaminant exceeded the relevant WHO value in Table 1. Annual exposure to multicontaminant air pollution was assessed for combinations of two or three contaminants and daily exposure was assessed for combinations of two, three, four or five contaminants (Table 2). To investigate the impact of urbanization on air pollution, we determined whether there was a correlation between the size of the urban population and the proportion of days in 2014 during which the concentration of specific contaminants exceeded WHO guideline values. For this analysis, cities were divided into five groups by population size, according to China's new urban size standard:^12^ (i) less than 0.5 million; (ii) 0.5 to less than 1 million; (iii) 1 to less than 5 million; (iv) 5 to less than 10 million; and (v) 10 million or more. The correlation between the population size and the percentage of days in 2014 with a high level of multicontaminant air pollution was determined using nonlinear regression analysis.

## Results

**Fig. 1:**
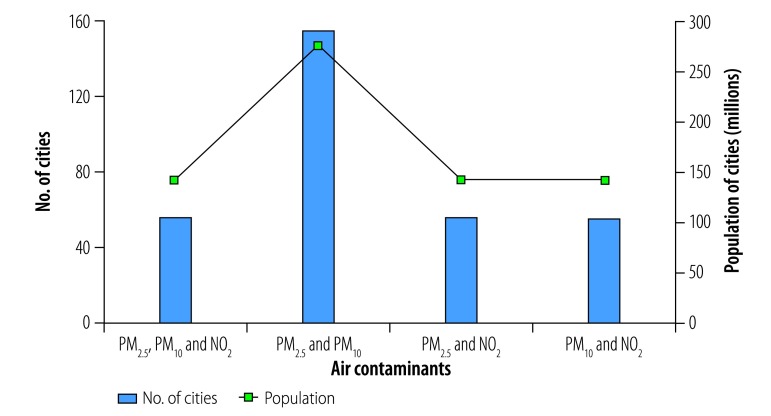
Cities with high mean annual air contaminant concentrations, by contaminant type, China, 2014

**Fig. 2:**
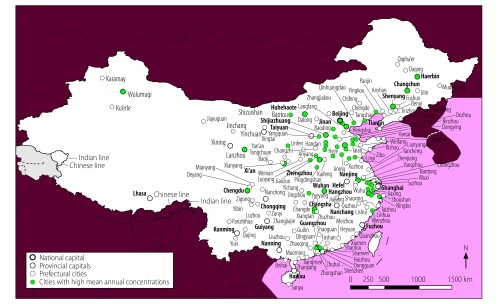
Locations of cities with high mean annual air multicontaminant concentrations, China, 2014

In total, 56 of the 155 cities analysed (36%) were exposed to mean annual concentrations of the contaminants PM_2.5_, PM_10_ and NO_2_ above WHO guideline values (Fig. 1). These cities had a combined population of 142 million out of a total study population of 276 million (i.e. 51%). In addition, all 155 cities were exposed to high annual concentrations of two-contaminant mixtures of PM_2.5_ and PM_10_ and 56 cities, with a total population of 142 million, were exposed to high annual concentrations of PM_2.5_ and NO_2_ and of PM_10_ and NO_2_. The cities with high annual multicontaminant exposure to either (i) PM_2.5_, PM_10_ and NO_2_; (ii) PM_2.5_ and NO_2_; or (iii) PM_10_ and NO_2_ were mainly located in east China, specifically in Hebei, Henan, Jiangsu, Shandong and Zhejiang Provinces and in the megacities of Beijing, Guangzhou, Shenzhen and Tianjin (Fig. 2).

NO_2_: nitrogen dioxide; PM_2.5_: fine particulate matter less than or equal to 2.5 μm in diameter; PM_10_: coarse particulate matter with a diameter between 2.5 and 10 μm.

Notes: The mean annual air contaminant concentration in 2014 was classed as high if it exceeded the World Health Organization guideline value (Table 1).

The dark blue circles represent cities with high mean annual air contaminant concentrations of either: (i) fine particulate matter less than or equal to 2.5 μm in diameter (PM_2.5_), coarse particulate matter with a diameter between 2.5 and 10 μm (PM_10_) and nitrogen dioxide (NO_2_); (ii) PM_2.5_ and NO_2_; or (iii) PM_10_ and NO_2_. The mean annual air contaminant concentration in 2014 was classed as high if it exceeded the World Health Organization guideline value (Table 1).

### Daily multicontaminant exposure

**Fig. 3:**
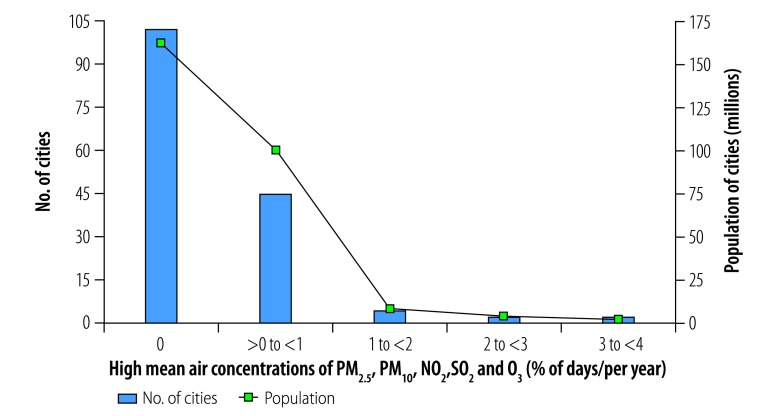
Cities with high mean daily air concentrations of PM_2.5_, PM_10_, NO_2_, SO_2_ and O_3_, by annual frequency, China, 2014

**Fig. 4:**
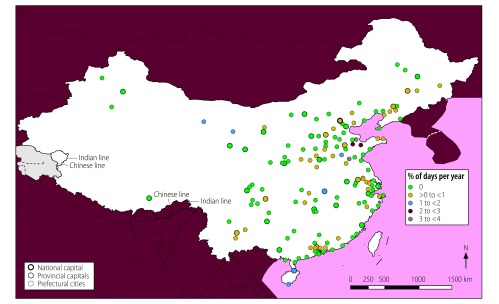
Locations of cities with high mean daily air concentrations of PM_2.5_, PM_10_, NO_2_, SO_2_ and O_3_, by annual frequency, China, 2014

Only two cities, Dongying and Linyi in Shandong Province, had mean daily concentrations of all five contaminants (i.e. PM_2.5_, PM_10_, SO_2_, O_3_ and NO_2_) above WHO guideline values for 11–15 days (3–4%) in 2014 (Fig. 3). Weifang and Zibo in Shandong Province were exposed to high daily concentrations of the five contaminants for 8–11 days (2–3%) in the year. Jining in Shandong Province, Wuhan in Hubei Province and Jiayuguan and Jinchang in Gansu Province were exposed to high daily concentrations for 4–8 days (1–2%; Fig. 4). Other cities had less than 4 days (1%) with high concentrations of all five contaminants.

NO_2_: nitrogen dioxide; O_3_: ozone; PM_2.5_: fine particulate matter less than or equal to 2.5 μm in diameter; PM_10_: coarse particulate matter with a diameter between 2.5 and 10 μm; SO_2_: sulfur dioxide.

Notes: The mean daily air contaminant concentration was classed as high if it exceeded the World Health Organization guideline value (Table 1). The study included 155 cities with a combined population of 276 million.

NO_2_: nitrogen dioxide; O_3_: ozone; PM_2.5_: fine particulate matter less than or equal to 2.5 μm in diameter; PM_10_: coarse particulate matter with a diameter between 2.5 and 10 μm; SO_2_: sulfur dioxide.

Note: The mean daily air contaminant concentration was classed as high if it exceeded the World Health Organization guideline value (Table 1).

**Fig. 5:**
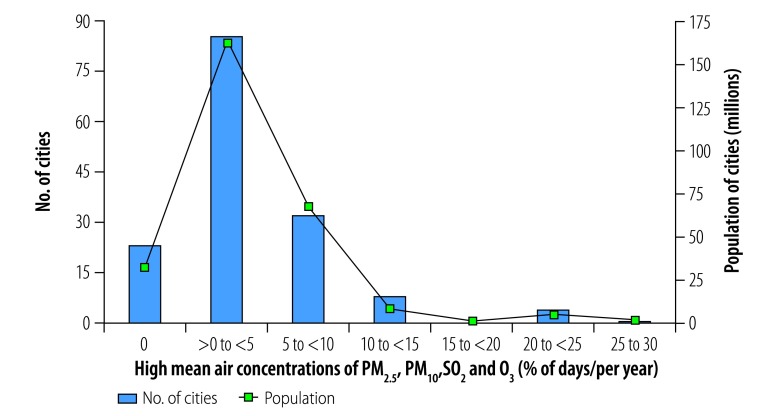
Cities with high mean daily air concentrations of PM_2.5_, PM_10_, SO_2_ and O_3_, by annual frequency, China, 2014

**Fig. 6:**
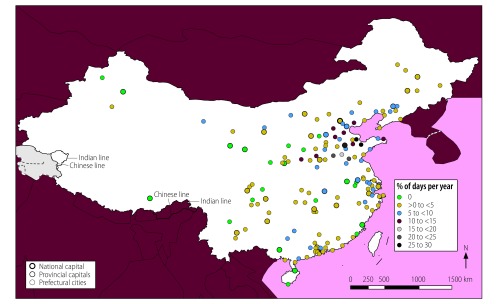
Locations of cities with high mean daily air concentrations of PM_2.5_, PM_10_, SO_2_ and O_3_, by annual frequency, China, 2014

**Fig. 7:**
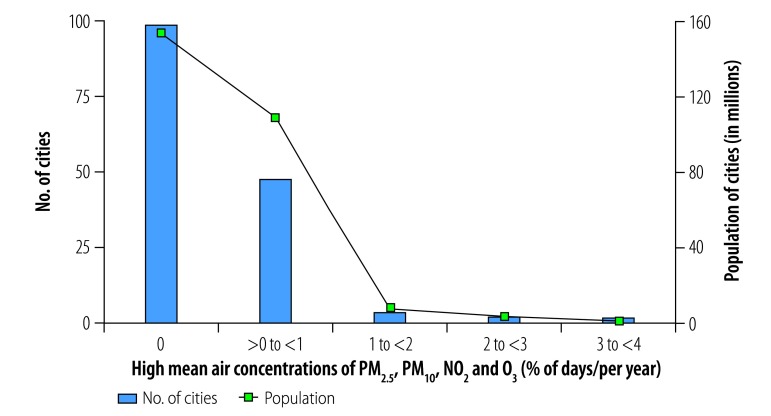
Cities with high mean daily air concentrations of PM_2.5_, PM_10_, NO_2_ and O_3_, by annual frequency, China, 2014

**Fig. 8:**
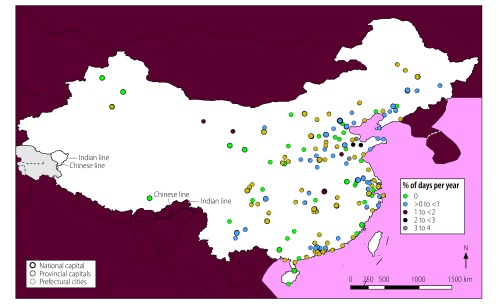
Locations of cities with high mean daily air concentrations of PM_2.5_, PM_10_, NO_2_ and O_3_, by annual frequency, China, 2014

Exposure to high mean daily concentrations of four contaminants was more common. In some locations, daily concentrations exceeded WHO guideline values for 73–110 days (20–30%) in 2014 for PM_2.5_, PM_10_, SO_2_ and O_3_ (Fig. 5) and for PM_2.5_, PM_10_, NO_2_ and SO_2_. The cities with the highest frequencies of exposure to high daily concentrations of the four contaminants PM_2.5_, PM_10_, SO_2_ and O_3_ were located in Shandong Province (Fig. 6; available at: http://www.who.int/bulletin/volumes/96/4/17-195560), whereas those with the highest frequencies of exposure to high daily concentrations of the four contaminants PM_2.5_, PM_10_, NO_2_ and SO_2_ were mainly located in Hebei and Shandong Provinces. High daily concentrations of other four-contaminant mixtures were rare: high daily concentrations of PM_2.5_, PM_10_, NO_2_ and O_3_ (Fig. 7 and Fig. 8; both available at: http://www.who.int/bulletin/volumes/96/4/17-195560), of PM_2.5_, O_3_, NO_2_ and SO_2_ and of PM_10_, O_3_, NO_2_ and SO_2_ were observed on less than 18 days (5%) in 2014 in most major Chinese cities.

O_3_: ozone; PM_2.5_: fine particulate matter less than or equal to 2.5 μm in diameter; PM_10_: coarse particulate matter with a diameter between 2.5 and 10 μm; SO_2_: sulfur dioxide.

Notes: The mean daily air contaminant concentration was classed as high if it exceeded the World Health Organization guideline value (Table 1). The study included 155 cities with a combined population of 276 million.

O_3_: ozone; PM_2.5_: fine particulate matter less than or equal to 2.5 μm in diameter; PM_10_: coarse particulate matter with a diameter between 2.5 and 10 μm; SO_2_: sulfur dioxide.

Note: The mean daily air contaminant concentration was classed as high if it exceeded the World Health Organization guideline value (Table 1).

NO_2_: nitrogen dioxide; O_3_: ozone; PM_2.5_: fine particulate matter less than or equal to 2.5 μm in diameter; PM_10_: coarse particulate matter with a diameter between 2.5 and 10 μm.

Notes: The mean daily air contaminant concentration was classed as high if it exceeded the World Health Organization guideline value (Table 1). The study included 155 cities with a combined population of 276 million.

NO_2_: nitrogen dioxide; O_3_: ozone; PM_2.5_: fine particulate matter less than or equal to 2.5 μm in diameter; PM_10_: coarse particulate matter with a diameter between 2.5 and 10 μm.

Note: The mean daily air contaminant concentration was classed as high if it exceeded the World Health Organization guideline value (Table 1).

**Fig. 9:**
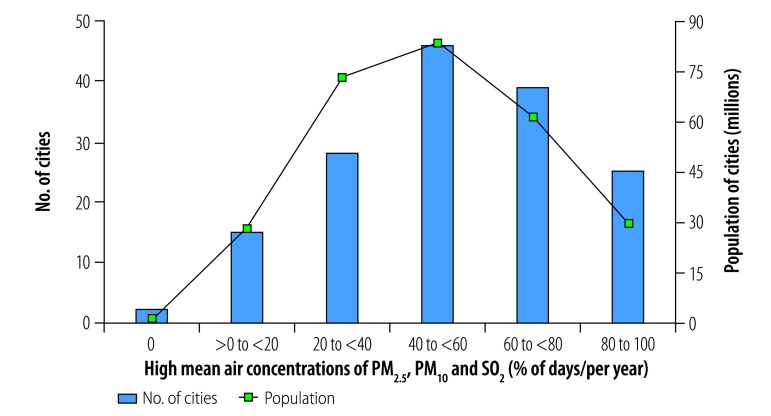
Cities with high mean daily air concentrations of PM_2.5_, PM_10_ and SO_2_, by annual frequency, China, 2014

**Fig. 10:**
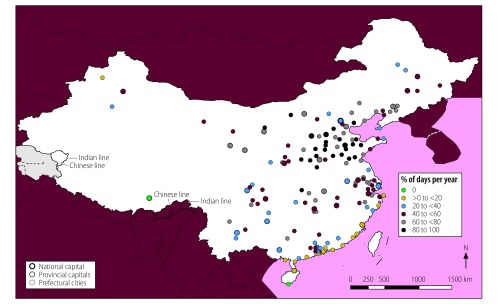
Locations of cities with high mean daily air concentrations of PM_2.5_, PM_10_ and SO_2_, by annual frequency, China, 2014

Exposure to high mean daily concentrations of the three contaminants PM_2.5_, PM_10_ and SO_2_ was even more common: 110 cities with a total population of 173 million were exposed to this level of air pollution for more than 146 days (40%) in 2014 (Fig. 9). Those cities were mainly located in east and central China, particularly in Hebei, Henan, Shandong and Shanxi Provinces (Fig. 10). In addition, exposure to high daily concentrations of mixtures of the following three-contaminant combinations were observed on 18–146 days (5–40%) in many cities: (i) PM_2.5_, PM_10_ and O_3_; (ii) PM_2.5_, O_3_ and SO_2_; (iii) PM_2.5_, PM_10_ and NO_2_; (iv) PM_2.5_, SO_2_ and NO_2_; (v) PM_10_, O_3_ and NO_2_; and (vi) PM_10_, O_3_ and SO_2_ (Table 3). However, high daily concentrations of the three-contaminant mixtures of (i) PM_2.5_, O_3_ and NO_2_, (ii) PM_10_, SO_2_ and NO_2_, and (iii) NO_2_, O_3_ and SO_2_ were observed on less than 18 days (5%) in 2014 in major Chinese cities.

PM_2.5_: fine particulate matter less than or equal to 2.5 μm in diameter; PM_10_: coarse particulate matter with a diameter between 2.5 and 10 μm; SO_2_: sulfur dioxide.

Notes: The mean daily air contaminant concentration was classed as high if it exceeded the World Health Organization guideline value (Table 1). The study included 155 cities with a combined population of 276 million.

PM_2.5_: fine particulate matter less than or equal to 2.5 μm in diameter; PM_10_: coarse particulate matter with a diameter between 2.5 and 10 μm; SO_2_: sulfur dioxide.

Note: The mean daily air contaminant concentration was classed as high if it exceeded the World Health Organization guideline value (Table 1).

**Table 3:** Frequency of high mean daily concentrations of air contaminants in 155 cities, by number of contaminants, China, 2014

No. of contaminants	Contaminant combinations with high mean daily concentrations^a^		
	High frequency (> 40% of days in 2014)	Medium frequency (5–40% of days in 2014)	Low frequency (< 5% of days in 2014)
**Four**	No cities	PM_2.5_, PM_10_, SO_2_ and O_3_ (46 cities)	PM_2.5_, PM_10_, NO_2_ and O_3_ (56 cities)
		PM_2.5_, PM_10_, NO_2_ and SO_2_ (25 cities)	PM_2.5_, O_3_, NO_2_ and SO_2_ (53 cities)
			PM_10_, O_3_, NO_2_ and SO_2_ (54 cities)
**Three**	PM_2.5_, PM_10_ and SO_2_ (147 cities)	PM_2.5_, PM_10_ and O_3_ (73 cities)	(PM_2.5_, O_3_ and NO_2_ (56 cities)
		PM_2.5_, O_3_ and SO_2_ (46 cities)	PM_10_, O_3_ and NO_2_ (57 cities)
		PM_2.5_, PM_10_ and NO_2_ (31 cities)	NO_2_, O_3_ and SO_2_ (55 cities)
		PM_2.5_, SO_2_ and NO_2_ (26 cities	
		PM_10_, SO_2_ and NO_2_ (27 cities)	
		PM_10_, O_3_ and SO_2_ (47 cities)	
**Two**	PM_2.5_ and PM_10_ (155 cities)	PM_2.5_ and O_3_ (76 cities)	O_3_ and NO_2_ (55 cities)
	PM_2.5_ and SO_2_ (147 cities)	PM_2.5_ and NO_2_ (33 cities)	
	PM_10_ and SO_2_ (147 cities)	PM_10_ and O_3_ (74 cities)	
		PM_10_ and NO_2_ (32 cities)	
		O_3_ and SO_2_ (47 cities)	
		NO_2_ and SO_2_ (28 cities)	

NA: not applicable; NO_2_: nitrogen dioxide; O_3_: ozone; PM_2.5_: fine particulate matter less than or equal to 2.5 μm in diameter; PM_10_: coarse particulate matter with a diameter between 2.5 and 10 μm; SO_2_: sulfur dioxide.

^a^ The mean daily air contaminant concentration was classed as high if it exceeded the World Health Organization guideline value (Table 1).

**Fig. 11:**
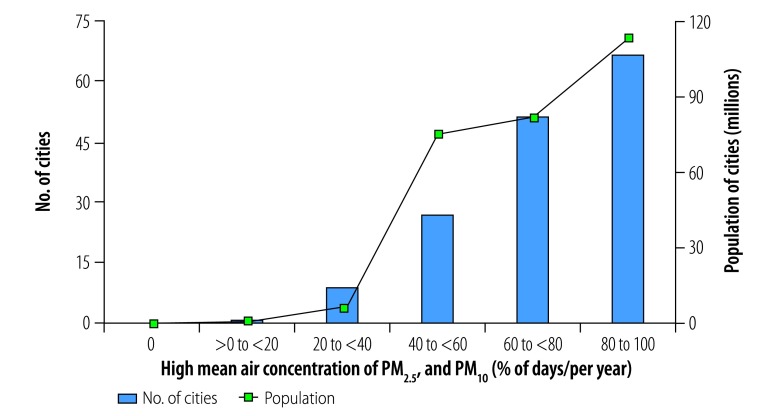
Cities with high mean daily air concentrations of PM_2.5_ and PM_10_, by annual frequency, China, 2014

**Fig. 12:**
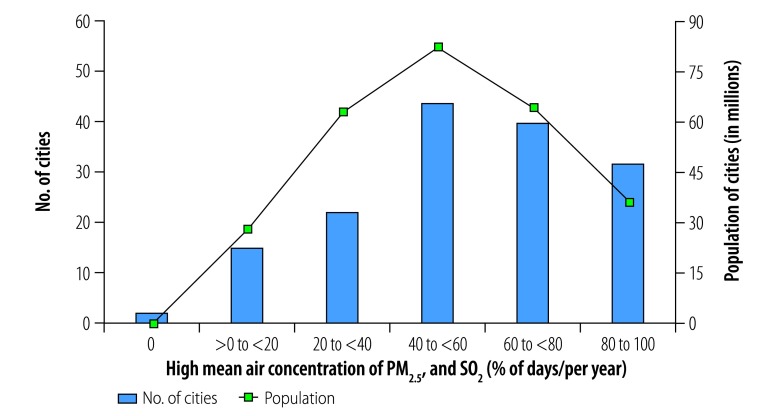
Cities with high mean daily air concentrations of PM_2.5_ and SO_2_, by annual frequency, China, 2014

**Fig. 13:**
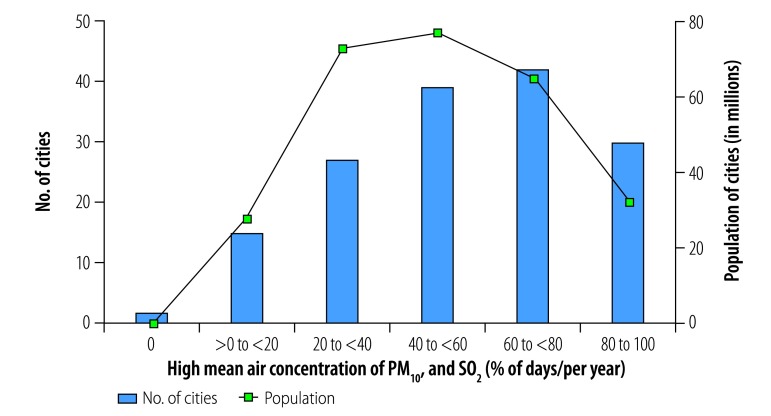
Cities with high mean daily air concentrations of PM_10_ and SO_2_, by annual frequency, China, 2014

**Fig. 14:**
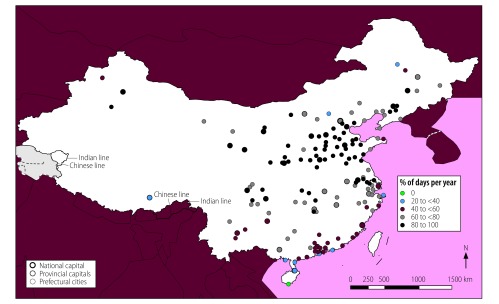
Locations of cities with high mean daily air concentrations of PM_2.5_ and PM_10_, by annual frequency, China, 2014

**Fig. 15:**
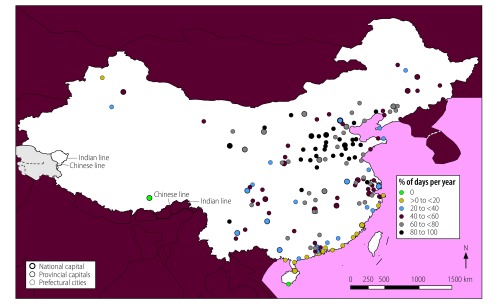
Locations of cities with high mean daily air concentrations of PM_2.5_ and SO_2_, by annual frequency, China, 2014

**Fig. 16:**
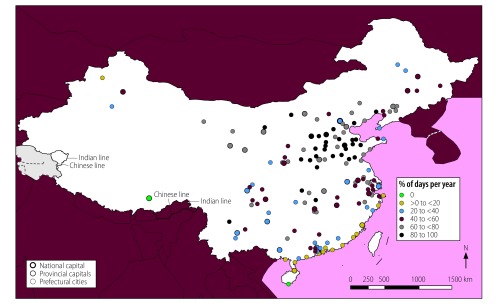
Locations of cities with high mean daily air concentrations of PM_10_ and SO_2_, by annual frequency, China, 2014

Exposure to high daily concentrations of two contaminants was extremely common: 145 cities with a total population of 269 million were exposed to mean daily concentrations of PM_2.5_ and PM_10_ above WHO guideline values for more than 146 days (40%) in 2014 (Fig. 11). High concentrations of the two contaminants PM_2.5_ and SO_2_ were also observed on more than 146 days (40%) in 116 cities with a total population of 184 million (Fig. 12; available at: http://www.who.int/bulletin/volumes/96/4/17-195560) and high concentrations of PM_10_ and SO_2_ were equally frequently observed in 111 cities with a total population of 175 million (Fig. 13; available at: http://www.who.int/bulletin/volumes/96/4/17-195560). The affected cities were mainly located in provinces in the east of China: Hebei, Henan, Shandong and Shanxi Provinces (Fig. 14, Fig. 15 and Fig. 16; all available at: http://www.who.int/bulletin/volumes/96/4/17-195560).

PM_2.5_: fine particulate matter less than or equal to 2.5 μm in diameter; PM_10_: coarse particulate matter with a diameter between 2.5 and 10 μm.

Notes: The mean daily air contaminant concentration was classed as high if it exceeded the World Health Organization guideline value (Table 1). The study included 155 cities with a combined population of 276 million.

PM_2.5_: fine particulate matter less than or equal to 2.5 μm in diameter; SO_2_: sulfur dioxide.

Notes: The mean daily air contaminant concentration was classed as high if it exceeded the World Health Organization guideline value (Table 1). The study included 155 cities with a combined population of 276 million.

PM_10_: coarse particulate matter with a diameter between 2.5 and 10 μm; SO_2_: sulfur dioxide.

Notes: The mean daily air contaminant concentration was classed as high if it exceeded the World Health Organization guideline value (Table 1). The study included 155 cities with a combined population of 276 million.

PM_2.5_: fine particulate matter less than or equal to 2.5 μm in diameter; PM_10_: coarse particulate matter with a diameter between 2.5 and 10 μm.

Note: The mean daily air contaminant concentration was classed as high if it exceeded the World Health Organization guideline value (Table 1).

PM_2.5_: fine particulate matter less than or equal to 2.5 μm in diameter; SO_2_: sulfur dioxide.

Note: The mean daily air contaminant concentration was classed as high if it exceeded the World Health Organization guideline value (Table 1).

PM_10_: coarse particulate matter with a diameter between 2.5 and 10 μm; SO_2_: sulfur dioxide.

Note: The mean daily air contaminant concentration was classed as high if it exceeded the World Health Organization guideline value (Table 1).

### Population size

**Fig. 17:**
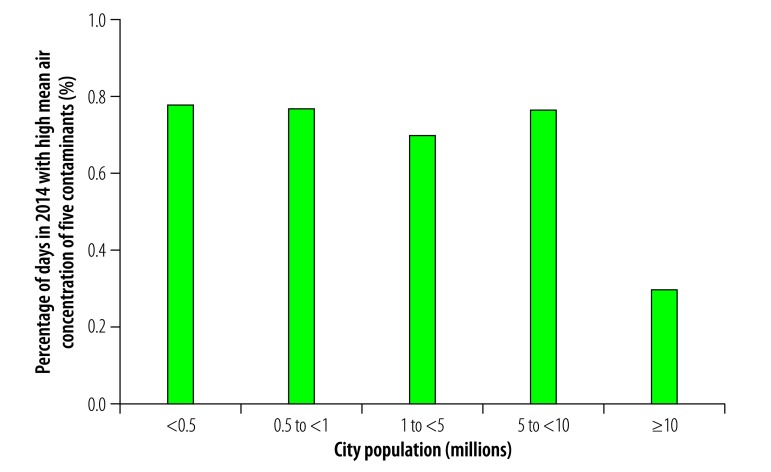
Cities with high mean daily air concentrations of five contaminants, by city population, China, 2014

Notes: The five contaminants were: (i) fine particulate matter less than or equal to 2.5 μm in diameter; (ii) coarse particulate matter with a diameter between 2.5 and 10 μm; (ii) nitrogen dioxide; (iv) sulfur dioxide; and (v) ozone. The mean daily air contaminant concentration was classed as high if it exceeded the World Health Organization guideline value (Table 1).

**Fig. 18:**
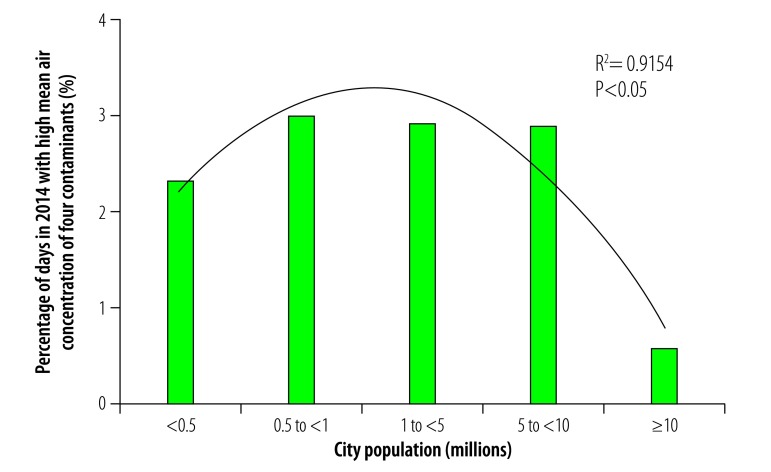
Cities with high mean daily air concentrations of four contaminants, by city population, China, 2014

Notes: The mean daily air contaminant concentrations of any four study contaminants were classed as high if they exceeded World Health Organization guideline values (Table 1). The five study contaminants were: (i) fine particulate matter less than or equal to 2.5 μm in diameter; (ii) coarse particulate matter with a diameter between 2.5 and 10 μm; (ii) nitrogen dioxide; (iv) sulfur dioxide; and (v) ozone. The equation for the regression line is *y* = –0.0042 *x^2^* + 0.0218 *x* + 0.0047.

**Fig. 19:**
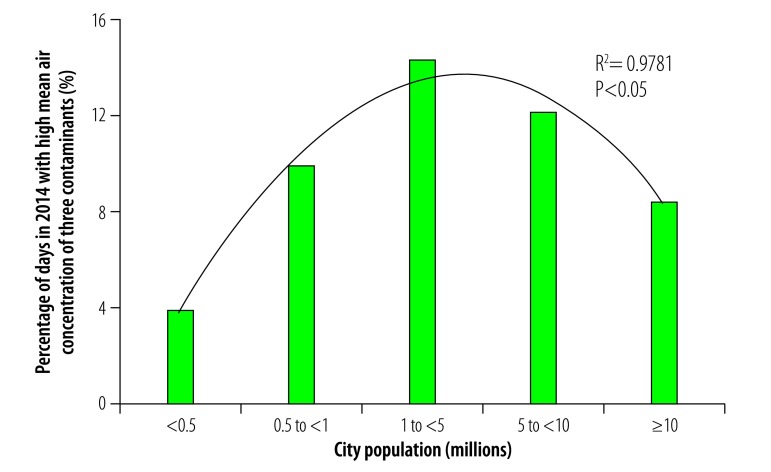
Cities with high mean daily air concentrations of three contaminants, by city population, China, 2014

Notes: The mean daily air contaminant concentrations of any three study contaminants were classed as high if they exceeded World Health Organization guideline values (Table 1). The five study contaminants were: (i) fine particulate matter less than or equal to 2.5 μm in diameter; (ii) coarse particulate matter with a diameter between 2.5 and 10 μm; (ii) nitrogen dioxide; (iv) sulfur dioxide; and (v) ozone. The equation for the regression line is *y* = –0.0184 *x^2^* + 0.1219 *x* – 0.0644.

**Fig. 20:**
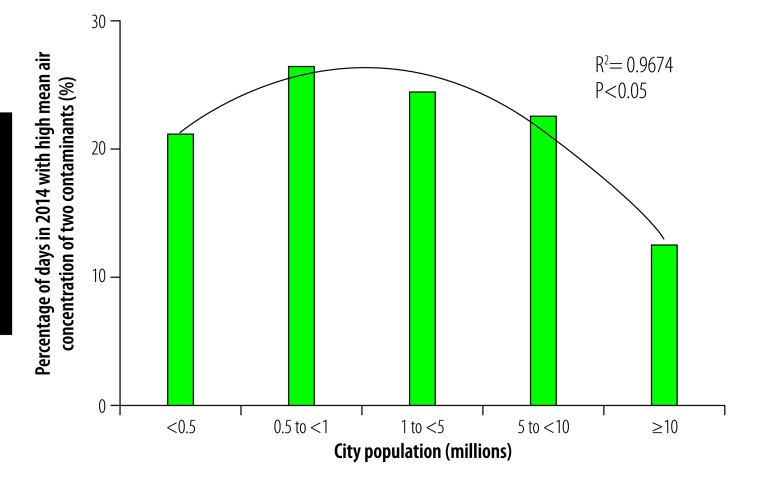
Cities with high mean daily air concentrations of two contaminants, by city population, China, 2014

Notes: The mean daily air contaminant concentrations of any two study contaminants were classed as high if they exceeded World Health Organization guideline values (Table 1). The five study contaminants were: (i) fine particulate matter less than or equal to 2.5 μm in diameter; (ii) coarse particulate matter with a diameter between 2.5 and 10 μm; (ii) nitrogen dioxide; (iv) sulfur dioxide; and (v) ozone. The equation for the regression line is *y* = –0.0216 *x^2^* + 0.1086 *x* + 0.1266.

In general, daily multicontaminant air pollution was less frequent in cities with a population greater than 10 million than in smaller cities in our study. For example, the proportion of days in 2014 during which the mean daily concentrations of all five contaminants exceeded WHO guideline values was not significantly affected by population size in cities with fewer than 10 million inhabitants but the portion was substantially lower in cities with a population greater than 10 million (Fig. 17). Similarly, the frequency of exposure to high mean daily concentrations of four contaminants was comparable among cities with populations ranging from 0.5 to 10 million but was lower in cities with a population less than 0.5 million or greater than 10 million (Fig. 18). This variation was also observed for high mean daily concentrations of three contaminants: the frequency was similar in cities with populations ranging from 0.5 to 10 million but lower in those with a population less than 0.5 million or greater than 10 million (Fig. 19). For exposure to high daily concentrations of two contaminants, there was no substantial variation in frequency among cities with a population less than 10 million, whereas the frequency was markedly lower in cities with a population greater than 10 million (Fig. 20). There was a significant inverse U-shaped relationship between the size of the urban population and the observed frequency of high mean daily concentrations of four contaminants (Fig. 18). In addition, there were inverse U-shaped relationships between population size and the frequency of high mean daily concentrations of three and two contaminants but the relationships were weaker (Fig. 19 and Fig. 20).

## Discussion

Although our study was based on data for only one year, it provides a snapshot of air pollution in major Chinese cities and demonstrates that multicontaminant air pollution was very common in 2014. These findings underscore the need to assess multiple air contaminant concentrations at the same time to obtain a more realistic picture of urban air quality and its potential impact on public health. Consequently, a change in air quality guidelines is required, with the establishment of guidelines on multicontaminant mixtures. The globally recognized, ambient air quality guidelines produced by WHO were designed to help reduce the health effects of air pollution in 1987. They were based on a review of the scientific evidence and its implications. The guidelines, which were updated in 1997 and 2005, now specify daily and annual limits for five major ambient air contaminants. In addition, some regions and countries have established their own air quality standards. For instance, the European Union, Japan and the United States of America were quick to update their air quality guidelines, whereas some middle- and low-income countries, e.g. China, established their own standards in response to high levels of pollution. However, all these guidelines and standards treat each contaminant in isolation or choose a single major contaminant as an indicator of air quality. For example, China uses an air quality index based on the maximum value of each individual contaminant's concentration to indicate air quality.^13^

Multicontaminant ambient air pollution is also important for public health research at both the urban and regional level. In the past, very little attention has been paid to multicontaminant exposure and research efforts have primarily focused on the health effects of individual contaminants. Initially, the reason for this focus was the difficulty of evaluating the medical effects of exposure to several contaminants. In addition, there was little understanding that multicontaminant ambient air pollution is common.^5^ However, without detailed research into the medical consequences of multicontaminant exposure, the disease burden will be underestimated. The influential Global Burden of Disease Study 2013 considered both ambient and household air pollution.^14^ Still, the only ambient air contaminants included were particulate matter and ozone, no consideration was given to other contaminants. We recommend that research into air pollution and its health effects should pay more attention to multicontaminant ambient air pollution, especially in middle- and low-income counties where current pollution levels are often higher than in high-income countries. In particular, by devoting attention to multicontaminant mixtures, researchers could raise public awareness of the complex nature of ambient air quality and stimulate greater interest in air pollution prevention.

As a result of rapid urbanization during the last century, more than half of the world's population now lives in cities.^15^ This rise in the urban population and the associated intensification of social and economic activity have had a substantial impact on urban air quality. Thus, urbanization and its effect on air quality are among the most important issues for achieving sustainable urban and regional development. Researchers have studied the relationship between urbanization and typical air contaminants in both developed and developing countries.^9^^,^^16^ For example, the concentration of the traditional air contaminant NO_2_ has been observed to increase exponentially with population size, though the value of the exponent varies between locations.^16^ In contrast, for PM_2.5_, the relationship between its concentration and urban population size is much more variable across continents and countries.^9^ In our study, we found an inverse U-shaped relationship between urban population size and the frequency of high daily concentrations of three contaminants, whereas other researchers have demonstrated no clear relationship. Furthermore, we discovered that a high level of multicontaminant air pollution was less common in cities with a population of more than 10 million than in smaller cities, which is contrary to general expectations that larger cities would be more polluted. The likely explanation is that large cities have implemented extensive environmental protection measures and that many polluting industries have been relocated to smaller cities.^3^ This observation casts new light on multicontaminant air pollution and its relationship to urbanization. We suggest that future research should pay more attention to the process of urbanization and its impact on multicontaminant ambient air pollution, particularly in middle- and low-income countries. Our findings highlight the varied pattern of multicontaminant air pollution in Chinese cities and confirm the view that pollution in developing countries should be expected to vary greatly across both time and space. Consequently, the results of this research should be relevant not only to China but also to other middle- and low-income countries facing similar challenges with multicontaminant air pollution.
